# Patient–Proxy Agreement Regarding Health-Related Quality of Life in Survivors with Lymphoma: A Propensity-Score Matching Analysis

**DOI:** 10.3390/cancers14030607

**Published:** 2022-01-25

**Authors:** Richard Huan Xu, Dong Dong

**Affiliations:** 1 Department of Rehabilitation Sciences, The Hong Kong Polytechnic University, Hong Kong SAR, China; richard.xu@polyu.edu.hk; 2 JC School of Public Health and Primary Care, The Chinese University of Hong Kong, Hong Kong SAR, China; 3 Shenzhen Research Institute, The Chinese University of Hong Kong, Shenzhen 518000, China

**Keywords:** lymphoma, health-related quality of life, patient–proxy agreement

## Abstract

**Simple Summary:**

This study investigated the difference in self- and proxy-reported Health-related quality of life (HRQoL), and their associations with sociodemographic and other health characteristics in a sample of Chinese lymphoma survivors. Propensity-score matching approach was used to reduce the bias by selecting a sample in which confounding factors were balanced between two patient groups. The findings show that, compared with proxy-reported patients, self-reported patients were more likely to indicate higher physical, role and emotional, but lower cognitive and social functioning. Further analysis confirmed that a statistically significant difference between self- and proxy-reported HRQoL was found when respondents reported being treated and having completed treatment. Additionally, regarding patients with different subtypes of lymphoma, the difference between patient self- and proxy-reported global HRQoL was not significant between different subtypes of lymphoma.

**Abstract:**

**Objective:** To assess the difference between lymphoma survivors’ self- and proxy-reported health-related quality of life (HRQoL) and its association with socioeconomic and health statuses. **Methods:** The data used in this study were obtained from a nationwide cross-sectional online survey in 2019. Information about participants’ demographics, health status and HRQoL were collected. The propensity-score matching (PSM) method was used to control the effect of potential confounders on selection bias. A chi-squared test, one-way analysis of variance, and multiple linear regression models were used to assess the relationship between HRQoL and response type adjusted to respondents’ background characteristics. **Results:** Out of the total 4400 participants, data of 2350 ones were elicited for analysis after PSM process. Patients’ self-reported outcomes indicated a slightly better physical, role and emotional functioning than proxy-reported outcomes. Regression analysis showed that patients, who were older, unemployed, and who received surgery, were more likely to report a lower HRQoL. Further analysis demonstrated that proxy-reported patients who had completed treatment were more likely to report a higher HRQoL than those who were being treated. **Conclusions:** Our study demonstrates that the agreement between self- and proxy-reported HRQoL is low in patients with lymphoma and the heterogeneities of HRQoL among patients with different types of aggressive NHL (Non-Hodgkin’s lymphoma) is large. Differences in self- and proxy-reported HRQoL should be considered by oncologists when selecting and deciding the optimal care plan for lymphoma survivors.

## 1. Introduction

Health-related quality of life (HRQoL), which reflects patients’ physical, mental, emotional, and social functioning over time, is increasingly identified as an important outcome for assessing the effectiveness of health and social care interventions [1,2]. Increasingly, studies have reported a significant association between socioeconomic status (SES) and HRQoL among cancer patients, where individuals with low income, education, and occupational position are more likely to report a poor HRQoL compared to their counterparts [3,4,5,6]. Lymphoma is a cancer that originates in the lymphatic system and can be categorised into two main types: Hodgkin’s lymphoma (HL) and non-Hodgkin’s lymphoma (NHL). Hodgkin’s lymphoma is a highly curable type and usually has good prognosis, whereas NHL has several subtypes and is more life-threatening [7]. Regarding lymphoma care, numerous studies have reported the influence of social inequalities, particularly on mortality and morbidity [8,9]. Although studies on the relationship between SES and HRQoL among lymphoma survivors have slowly increased in recent years [10,11,12], most investigations on this relationship were collected solely through patients’ self-reported data. In clinical practice, when cancer patients are in poor physical or mental health and are unable to respond, proxies are asked to report on the patient’s behalf [13]; however, the impact of proxy reports on HRQoL in surveys has rarely been assessed. A systematic review reported that survivors of childhood acute lymphoblastic leukemia and their parents’ sociodemographic and psychological factors were associated with HRQoL [14]. No evidence was provided about the effect of responses from different types of respondents on HRQoL and whether different responses were reshaped by the SES of lymphoma survivors.

According to Snow et al.’s definition, proxy-reported data refers to the data “collected from someone who speaks for a patient who cannot, will not, or is unavailable to speak for him or herself” [15]. In theory, the outcome reported by a true proxy should be no different from that of the patient; however, in effect, proxy responses have shown varied effects on the estimations of patients’ care experience and satisfaction [16]. Roydhouse et al. found that proxies indicated better healthcare experiences than patients diagnosed with incident colorectal or lung cancer [16]. However, Elliott et al. indicated that proxies tend to give more negative reports on health care than patients from the CAHPS MFFS survey [17]. Health-related quality of life is a relatively subjective concept, which is defined as individuals’ subjective disadvantage of being ill [18]. It assesses the health outcome not only from the perspective of individual health status, such as symptoms or side effects, but also includes the effect of many non-health-related aspects, such as family relationships and employment [19]. Evaluating the effectiveness of intervention on patients’ HRQoL requires the assessment of both the results of clinical tests and patients’ preferences, expectations, and experiences. However, proxy-reported data may affect the results of HRQoL estimations because of their different preferences and expectations regarding treatment and SES of patients [20], which might generate biased results and affect clinical decision-making. Therefore, the objective of this study was to assess the difference in patient self- and proxy-reported HRQoL and its association with SES and health status, using a population-based survey of patients with lymphomas in China.

## 2. Method

### 2.1. Study Design and Setting

A nationwide cross-sectional online survey was conducted to examine the socioeconomic and health statuses of patients with lymphoma in China between May and July 2019. The research team collaborated with “House086”, which is the largest national lymphoma patient organization, to recruit lymphoma survivors for this study. The inclusion criteria were as follow: (1) ≥18 years; (2) formally diagnosed with lymphoma; (3) able to read and write Chinese; (4) have no cognitive problems; (5) able to provide informed consent. All eligible members who met our criteria were invited to participate in the survey through House086’s internal social media groups. All interested members were contacted and requested to join a specific online “survey group”. Research investigators informed them about the objective of the study and guidelines for completing the online questionnaire. The survey was self-administered with the assistance from House086 staff. Informed consent was obtained on the first page of the questionnaire. Respondents could not begin the questionnaire until they had read the entire content about informed consent and clicked the ‘Agree’ button at the end of that page. As some patients might not be able to complete the questionnaire all by themselves due to poor health status, their main caregivers were recruited to complete the survey for them. Therefore, at the beginning of the survey, participants were required to indicate their identity as a patient or a caregiver. Thereafter, patients and caregivers were asked to complete different versions of the questionnaire, and their responses were coded as either self- or proxy-completed data.

### 2.2. Outcome Measures

The Chinese EORTC QLQ-C30 was used in this study. It is a non-preference-based patient-reported outcome measure, and showed good psychometrical properties in Chinese cancer populations [21]. It comprises 30 items for evaluating the generic HRQoL of cancer patients [22]. It has a global health status subscale, five functional subscales, and nine symptom subscales and items. The scores for each subscale were converted to a range between 0 and 100. For the global health and functional subscales, a higher sum score indicated a better status. For symptom subscales and items, a higher sum score indicated a worse status.

### 2.3. Covariates and Prospensity-Score Matching Approach

Propensity-score matching (PSM) is a quasi-experimental method to construct an artificial control group by matching each treated unit with a non-treated unit of similar characteristics. In this study, given differences of socioeconomic characteristics between self-reported patients and proxy-reported patients were significant. To control for the effect of potential confounders on selection bias, a propensity-score matched pair method with 1-to-1 nearest neighbor matching, without replacement, and a caliper of 0.01, was used. The multiple logistic regression model with covariates was used to estimate propensity scores to gauge the probability of participants from different groups. In this study, the unbalanced conditions between the two groups (self- and proxy-reported patients) were controlled by using PSM with covariate adjustment to ensure generating an equal number of matched pairs of participants having no differences in sex, age, educational level, family registration and subtype of lymphoma between self- and proxy-reported group.

### 2.4. Statistical Analyses

Descriptive statistics were used to summarize the respondent characteristics. All the variables were defined into four categories: ‘response type’, comprising two categories (self- and proxy-reported patients); ‘demographic variables’, comprising sex (male and female) and age groups (18–40, 41–60, ≥61); ‘SES’, comprising educational level (secondary and below, tertiary and above), family registry (rural resident and urban resident), marital status (single, married, divorced/widow(er)), family income (≤50,000 RMB per year, 50,001 RMB-100,000 RMB, ≥100,001 RMB), employment status (employed, unemployed, retired), and health insurance (Urban Employee Basic Medical Scheme, Urban Resident Basic Medical Scheme, New Rural Cooperative Medical Scheme, and Free Medical Scheme); and ‘treatment variables’, comprising treatment (chemotherapy, immunotherapy, radiotherapy, and surgery), duration of lymphoma, treatment status (never treated, treatment yet to start, being treated, treatment completed), and subtype of lymphoma (HL, aggressive NHL [A-NHL], and indolent NHL [I-NHL]). Three types of caregivers (patient’s adult child, spouse, and others) were considered, where others referred to patients’ parents, grandparents, relatives, friends, or others. A chi-squared test was used to compare the differences in respondent characteristics between the self- and proxy-reported patient subgroups.

The mean score and standard deviation of the QLQ-C30 subscales and items for all respondents were calculated and stratified by response type and proxy type. One-way analysis of variance (ANOVA) was used to examine group differences in the results of HRQoL between the self- and proxy-reported patients. Additionally, the mean scores and associated 95% confidence interval (95% CI) for QLQ-C30 subscales and items stratified by subtypes of lymphoma were calculated using ANOVA. Multiple linear regression models were used to assess the relationship between HRQoL and SES. Five models were developed. Model 1 assessed the relationship between HRQoL and response type adjusted for demographic, SES, and treatment variables; Model 2 assessed the relationship between HRQoL and response type adjusted for demographic and SES variables among patients who reported never receiving any kind of treatment; Model 3 assessed the relationship between HRQoL and response type adjusted for demographic, SES, and treatment (except for treatment status) variables among patients who reported not starting any treatment yet; Model 4 assessed the relationship between HRQoL and response type adjusted for demographic, SES, and treatment (except for treatment status) variables among patients who reported being treated; Model 5 assessed the relationship between HRQoL and response type adjusted for demographic, SES, and treatment (except for treatment status) variables among patients who completed the treatment within six months. All analyses were performed using R software [23], and the statistical significance level was set at *p* < 0.05.

## 3. Results

Table 1 indicates that a total of 4400 patients met the eligibility criteria, which comprised 2110 self-reported patients and 2290 proxy-reported patients. After the PSM, 1175 patients were assigned to the self-reported patient group and successfully matched with another 1175 patients in the proxy-reported comparison group. Approximately 60.3% were male, more than 60% were older than 40 years, and 72.9% were urban residents. Compared with proxy-reported patients, self-reported patients were highly employed, well-paid, highly covered by UEBS, and were being treated. Approximately 14.3%, 53.7% and 32% of patients reported having HL, A-NHL and I-NHL, respectively.

The results of the QLQ-C30 stratified by response type are presented in Table 2. Self-reported patients showed a significantly higher score on the physical, role, emotional, and cognitive function subscales than proxy-reported patients. Proxy-reported patients demonstrated a significantly lower score on fatigue (self-report = 41.03, proxy-report = 46.66, *p* < 0.001), nausea/vomiting (patient-report = 10.24, proxy-report = 13.9, *p* < 0.001), pain (patient-report = 19.67, proxy-report = 23.25, *p* < 0.001), dyspnea (patient-report = 38.64, proxy-report = 43.8, *p* < 0.001), appetite loss (patient-report = 20.6, proxy-report = 27.89, *p* < 0.001), and constipation (patient-report = 15.57, proxy-report = 19.23, *p* < 0.001) symptom subscales. Caregivers who are patients’ children tended to report that patients had better social functions, and less insomnia and constipation, than other types of caregiver.

**Figure 1:**
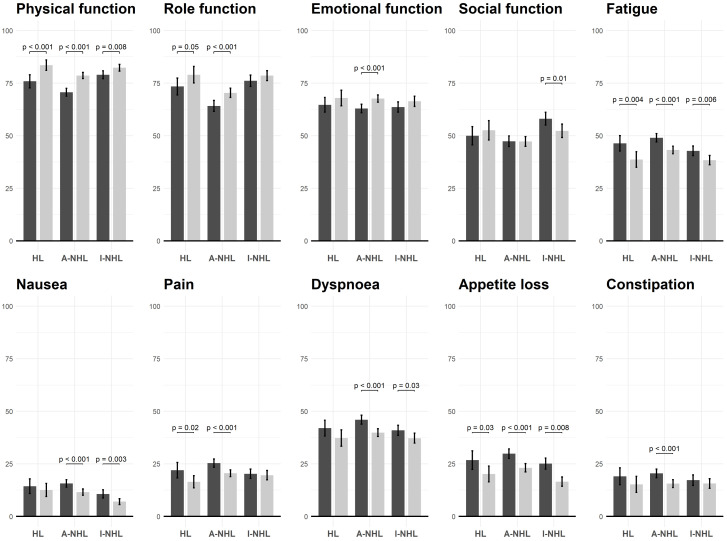
Significant difference of QLQ-C30 items, stratified by response type and lymphoma types. Dark and light grey indicates proxy-reported and self-reported patients, respectively; HL: Hodgkin’s lymphoma, A-NHL: Aggressive Non-Hodgkin’s lymphoma, I-NHL: Indolent Non-Hodgkin’s lymphoma.

Figure 1 presents the statistically significant difference between self- and proxy-reported QLQ-C30 subscales and items stratified by subtypes of lymphoma. Self-reported HL patients tended to report a better status on physical and role function, fatigue, pain, and appetite loss than proxy-reported HL patients. Self-reported A-NHL patients tended to report a better status on physical, role and emotional function, and fatigue, nausea, pain, dyspnea, appetite loss, and constipation than proxy-reported A-NHL patients. Regarding I-NHL survivors, self-reported patients were more likely to demonstrate a better physical and social function and fewer fatigue, nausea, dyspnea, and appetite loss symptoms. Comparisons for all items of the QLQ-C30 are presented in Table S1 (Supplementary Material).

Multiple linear regression analysis indicated that proxies reported a higher physical (coefficient [b] = 4.73, [95% CI 2.36, 7.1]), but lower cognitive (b = −3.98, [95% CI −6.53, −1.42]), and social HRQoL (b = −5.09, [95% CI −8.62, −1.55]), than patients when adjusted for all demographic, SES, and health condition variables (Table 3). Patients who were unemployed and being treated reported a lower HRQoL than patients who were employed and completed treatment in all five models, respectively. Middle-aged patients tended to report poorer physical, role, and social functioning than young patients. Patients with higher family income were more likely to report better physical, emotional, cognitive and social functioning. Stratified analyses showed that the difference between self-reported and proxy-reported physical functioning was statistically significant for those who were being treated or were given complete treatment. However, in terms of role and emotional functioning, for patients who were being treated or were given complete treatment, the difference between self-reported and proxy-reported outcomes was not significant. Proxy-reported cognitive and social functioning was significantly higher than self-reported ones only for patients who completed treatment (Table S2, Supplementary Materials).

## 4. Discussion

This study investigated the difference in self- and proxy-reported HRQoL, and their associations with SES and other health characteristics in a sample of Chinese lymphoma survivors. PSM approach was used to reduce the bias by selecting a sample in which confounding factors were balanced between two patient groups. We found that, compared with proxy-reported patients, self-reported patients were more likely to indicate higher physical, role and emotional, but lower cognitive and social (statistically non-significant) functioning. However, after adjusting patients’ demographics, SES, and treatment status, the difference in role and emotional functioning was not significant. Further analysis confirmed that a statistically significant difference between self- and proxy-reported HRQoL was found when respondents reported being treated and having completed treatment. Additionally, regarding patients with different subtypes of lymphoma, the difference between patient self- and proxy-reported global HRQoL was not significant between HL, A-NHL and I-NHL. However, within the group of patients with A-NHL, the differences between self- and proxy-reported HRQoL were significantly different in terms of four functions and six symptoms of the QLQ-C30.

Overall, our findings are in line with those of previous studies that showed a mismatch between the preference between self- and proxy-reported HRQoL in cancer survivors. For example, Jones et al. indicated that, in a Canadian sample, patients who received palliative cancer care tended to report a higher HRQoL compared to their caregivers’ reports [24]. Akin and Durna demonstrated that cancer patients are more likely to report a higher level of pain, depression, anxiety, drowsiness, and loss of well-being than their caregivers in Turkey [25]. However, several studies targeting pediatric cancer patients reported a different result, with parents generally reporting a poorer HRQoL or more psychological problems for patients than self-reporting children [13,26]. One possible explanation for this discrepancy may be the study population; that is, no children or adolescents were included in our study. As similar studies have rarely been conducted in China, further investigations of the difference between self- and proxy-reported HRQoL targeting on young cancer patients are needed.

Regarding proxy-reported data, patients’ children reported a significantly higher social functioning, but fewer symptoms such as insomnia and constipation than that reported by patients’ spouses, which were similar to patients’ self-reports. For most studies on HRQoL, comparisons of patients’ HRQoL reported by different types of caregivers were rarely conducted. Several studies confirmed that the proxy–patient relationship may play an important role in measuring patients’ HRQoL. However, the findings were mixed, with some studies indicating that the HRQoL reported by spouses are more reliable [17,20], whereas others have drawn a different conclusion [27]. Our study contributed knowledge regarding the quantification of the heterogeneity of HRQoL rated by different caregivers of patients with lymphomas. A possible explanation might be that, as compared to the other types of caregivers, spouses are more engaged in caring for the patients’ daily life needs, which improves their understanding of the patients’ experiences and feelings; thus, spouses are able to provide a similar HRQoL estimation as the patients themselves.

The highest level of mismatch of self- and proxy-reported HRQoL in our study was observed between patients who reported having completed treatment within six months and those being treated. No studies have discussed the differences in self- and proxy-reported health or HRQoL in patients at different stages of their cancer journey. A possible explanation of such a difference in HRQoL might be due to the re-balancing of the caregiver’s life and their feelings of relief from the distress of practical and emotional challenges coming to an end with the completed treatment [28]. A previous systematic review indicated that a substantial number of caregivers experienced stress, anxiety, or another emotional burden as a result of patient care, and they may feel relief when the treatment is completed; this feeling may affect their judgment on patients’ HRQoL [29]. Another explanation is that the lower HRQoL of self-reported patients compared to proxy reports was due to the patients’ negative emotions generated by an unexpected clinical outcome or one that was below the patients’ treatment expectations [30].

Regarding HRQoL reported by patients with different subtypes of lymphoma, proxy-reported HRQoL was poorer for patients with A-HNL than HL and I-NHL in terms of the four functions and six symptoms of the QLQ-C30. This difference between self- and proxy-reported HRQoL has never been discussed previously and is difficult to explain in this study. One explanation might be that despite aggressive NHL being a fast-growing disease, many patients can be treated and the prognosis is usually satisfying [31]. Thus, patients in our sample were more optimistic than their caregivers about the outcomes of treatment and thus resulted in a higher HRQoL reported by the patients than by the caregivers. Another reason might be that age is known as an important prognostic factor in A-NHL. Individuals who develop NHL before the age of 60 do better than those over 60 [32]. In our sample, more than 60% of the patients were younger than 60 years. Given the heterogeneity of HRQoL that were reported within aggressive lymphomas (Table S3, Supplementary Materials), further investigations are needed to clarify this issue.

Financial hardships resulting from increased medical expenses might be another factor affecting the relationship between self- and proxy-reported HRQoL [33,34]. Our previous study found a significant association between high subjective financial distress and low HRQoL in lymphoma patients [35]. During cancer treatment, the increased medical costs and related unpredictable outcomes might bring similar negative emotions for both patients and their caregivers [33], which, in turn, would narrow the gap in HRQoL estimations reported between patients and caregivers. However, in the recovery journey, with the improved physical and mental health statuses and the different expectations of life purpose [36], hope [37], and connectedness [38], the differences between patients’ and caregivers’ experiences increase. Our study found that both self- and proxy-reported patients with a high family income tend to report a high physical, cognitive, and social functioning for those who completed the treatment than those being treated. However, in this study, we did not collect the information about the length and type of treatment that patients had completed, and hence, some heterogeneities might not have been detected. Additionally, no causal conclusion can be drawn due to the cross-sectional design in which all the data were collected at one time point; and HRQoL may have varied over time with the change in patients’ functioning and symptoms.

This study had several strengths. First, our study uses PSM approach to select a comparable sample from a large (*n* = 4400) population-based cohort of lymphoma patients to assess the HRQoL in China. Our sample had good representativeness, which included patients from nearly all the provinces of China, including both developed and underdeveloped regions, and ages between 18 and 85 years. It provided baseline HRQoL data for lymphoma patient groups. Second, this study was the first of its kind to compare self- and proxy-reported HRQoL among patients with different subtypes of lymphomas worldwide. The results provide important implications for future assessment of the effectiveness of clinical, healthcare, and social care interventions to improve the health and well-being of this patient group, and it may be used as a part of quality assessment for oncology clinicians to base their findings on diverse perspectives.

There were some limitations to this study. First, the proxy-reported HRQoL data were reported by caregivers of other lymphoma patients, rather than the caregivers of the self-reported patients in our sample. Compared with proxy-reported patients, self-reported patients were young, better educated, and had relatively mild lymphoma-related health problems. Despite the PSM approach being used to reduce the bias due to several confounding variables, selection bias might have existed. Second, since the data used in this study were obtained from an online survey, interview and selection biases cannot be ignored as patients who are inactive Internet users may have been excluded from the survey. Third, information about patients’ lymphoma-related symptoms and clinical indicators were not collected in the survey, which might have affected the validity of our findings. Last, we did not collect the caregivers’ personal information in the survey, which might have led to some information and selection biases when assessing the agreement between self- and proxy-reported HRQoL.

## 5. Conclusions

Our study demonstrated that the agreement between self- and proxy-reported HRQoL is low among patients with lymphoma; self-reported patients tended to report a better HRQoL than proxy-reported patients, and the heterogeneities of HRQoL among patients with different types of aggressive NHL is large. Although our sample selection was based on PSM, the potential reporting error should not be ignored and needs further investigation. Our findings suggest that when using HRQoL to assess the effectiveness of lymphoma-related interventions, the type of reporter may have a significant impact on reports of changes in HRQoL. Additionally, different stages of the cancer treatment journey can affect the impact to some extent. Medical professionals should be careful when selecting the optimal care plan to meet patients’ preferences and needs in clinical practice.

## Figures and Tables

**Table 1:** Respondent’s characteristics.

	Before PSM (*n* = 4400)			After PSM (*n* = 2350)			
	Patient *n* = 2110	Proxy *n* = 2290	*p*-Value	Overall	Patient *n* = 1175	Proxy *n* = 1175	*p*-Value
**Survival years, mean (range)**	1.37 (1–35)	1.37 (1–30)	0.99	1.34 (1–35)	1.38 (1–35)	1.31 (1–13)	0.99
**Sex**							
Male	1077 (51)	1373 (59.96)	<0.001	1421 (60.3)	708 (60.3)	713 (60.3)	0.88
Female	1033 (49)	917 (40.04)		929 (39.7)	467 (39.7)	462 (39.7)	
**Age**							
18–40	1146 (54.3)	459 (20)	<0.001	919 (39.1)	460 (39.1)	459 (39.1)	0.99
41–60	810 (38.4)	1024 (44.7)		1143 (48.6)	571 (48.6)	572 (48.6)	
≥61	154 (7.3)	807 (35.2)		288 (12.3)	144 (12.3)	144 (12.3)	
**Educational level**							
Secondary or below	772 (36.6)	1646 (71.9)	<0.001	1173 (49.9)	587 (50)	586 (49.9)	0.98
Tertiary or above	1338 (63.4)	644 (28.1)		1177 (50.1)	588 (50)	589 (50.1)	
**Sub-types**							
HL	426 (20.2)	218 (9.5)	<0.001	335 (14.3)	162 (13.8)	173 (14.7)	0.81
A-NHL	977 (40)	1467 (60)		1263 (53.7)	634 (54)	629 (53.5)	
I-NHL	707 (53.9)	605 (46.1)		752 (32)	379 (32.2)	373 (31.7)	
**Family registry**							
Urban resident	1502 (71.1)	1430 (62.4)	<0.001	1711 (72.9)	853 (73.1)	858 (72.7)	0.84
Rural resident	597 (28.3)	854 (37.3)		636 (27.1)	320 (26.9)	316 (27.3)	
**Marital status**							
Single	340 (16.1)	131 (5.7)	<0.001	245 (10.4)	79 (6.7)	50 (4.3)	0.06
Married	1605 (76.1)	2014 (87.9)		1976 (84.1)	980 (83.4)	996 (84.7)	
Divorce/widow(er)	165 (7.8)	145 (6.3)		129 (5.5)	116 (9.9)	129 (11)	
**Family income per year**							
≤50,000 RMB	696 (44.1)	813 (45.3)	0.55	540 (36.2)	294 (39.4)	246 (33.1)	0.02
50,001~100,000 RMB	637 (40.4)	724 (40.4)		319 (21.4)	142 (19)	177 (23.8)	
≥100,001 RMB	245 (15.5)	256 (14.3)		631 (42.3)	310 (41.6)	321 (43.1)	
**Employed status**							
Employed	1055 (50)	837 (36.6)	<0.001	1117 (47.5)	612 (52.1)	505 (43)	<0.001
Non-employed	764 (36.2)	696 (30.4)		735 (31.3)	341 (29)	394 (33.5)	
Retired	291 (13.8)	757 (33)		298 (21.2)	222 (18.9)	272 (23.5)	
**Health insurance**							
UEBS	1137 (63.2)	957 (47.7)	<0.001	1175 (59.2)	629 (63.9)	546 (54.5)	<0.001
URBS	214 (11.9)	341 (17)		294 (14.8)	117 (11.9)	177 (17.7)	
NRMS	317 (17.6)	610 (30.3)		364 (18.3)	159 (16.2)	205 (20.5)	
FMS	130 (7.2)	102 (5.1)		153 (7.7)	79 (8)	74 (7.4)	
**Chemotherapy, Yes**	1833 (92.2)	2002 (91.5)	0.4	2038 (91.6)	1017 (91.7)	1021 (91.4)	0.8
**Immunotherapy, Yes**	776 (36.8)	858 (37.5)	0.9	893 (40.1)	449 (39.7)	444 (40.5)	0.72
**Radiotherapy, Yes**	498 (23.6)	361 (15.8)	<0.001	476 (21.4)	244 (22)	232 (20.8)	0.48
**Surgery, Yes**	296 (14)	319 (13.9)	0.77	338 (15.2)	161 (14.5)	177 (15.8)	0.38
**Treatment status**							
Never treated	122 (5.8)	102 (4.5)	<0.001	124 (5.3)	58 (4.9)	66 (5.6)	<0.001
Treatment not started yet	154 (7.3)	112 (4.9)		150 (6.4)	57 (4.9)	93 (7.9)	
Being treated	887 (42)	1281 (55.9)		1146 (48.8)	640 (54.5)	506 (43.1)	
Treatment completed	947 (44.9)	795 (34.7)		930 (39.6)	420 (35.7)	510 (43.4)	

UEBS: Urban Employee Basic Medical Scheme. URBS: Urban Resident Basic Medical Scheme. NRMS: The New Rural Cooperative Medical Scheme. FMS: Free Medical Scheme. HL: Hodgkin’s lymphoma. A-NHL: Aggressive Non-Hodgkin’s lymphomas. I-NHL: Indolent Non-Hodgkin’s lymphomas.

**Table 2:** Patients’ QoL measured by QLQ-C30 and stratified by response type.

	Mean (Standard Deviation)						
	Type of Response			Type of Caregiver			
	Self- Reported	Proxy- Reported	*p*-Value	Children	Spouse	Others	*p*-Value
***n***	1175	1175		411 (35)	529 (45)	235 (20)	
**Global health status**	61.85 (22.67)	60.48 (23)	0.14	62.35 (20.44)	61.17 (23.02)	62.52 (25.46)	0.93
**Functional scales**							
Physical	80.5 (17.56)	74.08 (22.3)	<0.001	75.16 (20.58)	72.42 (23.61)	74.52 (24.19)	0.11
Role	74.23 (26.97)	69.33 (30.52)	<0.001	69.55 (31.03)	68.65 (30.25)	70.5 (30.3)	0.81
Emotional	67.28 (23.26)	63.43 (24.94)	<0.001	63.3 (24.28)	62.85 (25.37)	64.72 (25.7)	0.39
Cognitive	75.42 (20.25)	77.28 (21.63)	0.03	76.84 (21.94)	76.64 (21.09)	79.36 (22.24)	0.16
Social	49.63 (30.75)	51.51 (31.32)	0.24	55.03 (30.58)	49.02 (31.02)	49.15 (32.71)	0.003
**Symptom scales**							
Fatigue	41.03 (23.12)	46.66 (24.58)	<0.001	46.9 (24.5)	47.64 (24.1)	44.02 (25.69)	0.24
Nausea/Vomiting	10.24 (17.82)	13.9 (22.18)	<0.001	12.45 (20.16)	14.18 (22.28)	15.82 (25.07)	0.06
Pain	19.67 (20.89)	23.25 (24.16)	<0.001	25.02 (24.7)	21.99 (22.95)	22.98 (25.72)	0.18
**Single items**							
Dyspnea	38.64 (23.85)	43.8 (26.05)	<0.001	44.28 (25.64)	45.12 (26.47)	40 (25.56)	0.09
Insomnia	30.27 (28.06)	30.35 (29.85)	0.94	35.36 (31.81)	28.42 (28.37)	25.96 (28.45)	<0.001
Appetite Loss	20.6 (24.56)	27.89 (28.17)	<0.001	28.71 (27.7)	26.84 (27.41)	28.79 (30.63)	0.85
Constipation	15.57 (23.68)	19.23 (26.01)	<0.001	21.41 (27.37)	17.64 (24.44)	19.01 (26.83)	0.04
Diarrhea	12.91 (19.77)	12.79 (21.09)	0.89	11.35 (19.08)	13.42 (21.67)	13.9 (22.97)	0.11

**Table 3:** Relationship between HRQoL and responded type adjusted by socioeconomic characteristics and health status.

	Coefficient (95% Confidence Interval)				
	Model 1 Physical Model	Model 2 Role Model	Model 3 Emotional Model	Model 4 Cognitive Model	Model 5 Social Model
Proxy-reported patients	4.73 (2.36, 7.1) ***	2.25 (−1.12, 5.61)	1.46 (−1.39, 4.32)	−3.98 (−6.53, −1.42) **	−5.09 (−8.62, −1.55) **
Female	−3.72 (−6.06, −1.38) **	−0.07 (−3.4, 3.25)	−2.28 (−5.1, 0.54)	−3.86 (−6.38, −1.33) **	0.06 (−3.43, 3.55)
41–60	−3.58 (−6.36, −0.79) *	−4.47 (−8.42, −0.52) *	−2.13 (−5.49, 1.22)	−2.65 (−5.65, 0.36)	−6.17 (−10.32, −2.02) **
≥61	−8.59 (−14.11, −3.07) **	−4.13 (−11.95, 3.7)	3.15 (−3.49, 9.8)	0.48 (−5.46, 6.43)	1.77 (−6.45, 9.99)
Tertiary and above	−2.44 (−5.17, 0.29)	−3.29 (−7.16, 0.58)	−1.11 (−4.39, 2.17)	−2.42 (−5.36, 0.52)	−3.61 (−7.67, 0.45)
Rural resident	−0.41 (−3.71, 2.89)	−4.18 (−8.86, 0.5)	0.54 (−3.44, 4.51)	2.25 (−1.3, 5.81)	−1.65 (−6.57, 3.26)
Married	0.97 (−4.11, 6.04)	3.34 (−3.86, 10.54)	−3.22 (−9.33, 2.89)	1.5 (−3.97, 6.97)	2.21 (−5.36, 9.77)
Divorce/widow(er)	3.54 (−2.8, 9.89)	7.98 (−1.02, 16.98)	0 (−7.64, 7.63)	3.33 (−3.51, 10.16)	1.08 (−8.37, 10.54)
50,001~100,000	5.72 (2.46, 8.97) ***	2.27 (−2.34, 6.89)	6.81 (2.9, 10.73) ***	5.68 (2.17, 9.19) **	11.52 (6.66, 16.37) ***
≥100,001	3.45 (0.8, 6.1) *	1.75 (−2, 5.5)	2.82 (−0.36, 6.01)	1.44 (−1.41, 4.29)	8.22 (4.27, 12.16) ***
Non-employed	−4.19 (−6.93, −1.44) **	−6.93 (−10.82, −3.03) ***	−4.32 (−7.62, −1.01) *	−4.88 (−7.84, −1.93) **	−5.98 (−10.07, −1.89) **
Retired	0.57 (−3.49, 4.63)	3.58 (−2.18, 9.34)	0.68 (−4.21, 5.57)	−2.97 (−7.34, 1.41)	1.18 (−4.87, 7.23)
URBS	1.46 (−3.69, 6.61)	0.98 (−6.32, 8.29)	3.27 (−2.93, 9.47)	1.84 (−3.7, 7.39)	4.36 (−3.31, 12.03)
NRCS	0.85 (−2.61, 4.3)	3.38 (−1.52, 8.28)	1.06 (−3.09, 5.22)	−0.73 (−4.45, 2.99)	0.78 (−4.36, 5.93)
FMS	2.74 (−1.04, 6.52)	5.15 (−0.21, 10.52)	2 (−2.55, 6.55)	−0.54 (−4.62, 3.53)	−0.81 (−6.45, 4.82)
Duration	0.09 (−0.64, 0.82)	0.51 (−0.53, 1.55)	0.02 (−0.86, 0.9)	−0.06 (−0.85, 0.72)	0.39 (−0.7, 1.48)
Chemotherapy, Yes	−2.66 (−7.19, 1.87)	−6.35 (−12.78, 0.08)	−4.18 (−9.63, 1.28)	−4.17 (−9.05, 0.72)	−6.52 (−13.27, 0.24)
Immunotherapy, Yes	1.32 (−1.04, 3.67)	2.12 (−1.21, 5.46)	0.13 (−2.7, 2.96)	0.45 (−2.08, 2.99)	0.37 (−3.13, 3.88)
Radiotherapy, Yes	1.17 (−1.54, 3.88)	3.75 (−0.09, 7.6)	0.12 (−3.14, 3.38)	−1.37 (−4.28, 1.55)	3.11 (−0.92, 7.15)
Surgery, Yes	−3.68 (−6.78, −0.59) *	−5.34 (−9.73, −0.95) *	−3.39 (−7.12, 0.33)	−1.81 (−5.15, 1.52)	−5.4 (−10.02, −0.79) *
Being treated	−11.42 (−13.72, −9.12) ***	−21.81 (−25.07, −18.54) ***	−12.62 (−15.39, −9.85) ***	−6.98 (−9.46, −4.5) ***	−16.82 (−20.25, −13.39) ***

Reference group: patient, male, 18–40 years, secondary or below educational level, urban residents, single, family income ≤ 50,000 RMB, active employed, UEBS (Urban Employer Basic Medical Scheme), no chemotherapy, no immunotherapy, no radiotherapy, no surgery, treatment completed. * *p* < 0.05; ** *p* < 0.01; *** *p* < 0.001. URBS = Urban Resident Basic Medical Scheme. NRCS = New Rural Cooperative Medical Scheme. FMS = Free Medical Scheme.

## Data Availability

Derived data supporting the findings of this study are available from the corresponding author on reasonable request.

## Supplementary Materials

The following supporting information can be downloaded at: https://www.mdpi.com/article/10.3390/cancers14030607/s1, Table S1: Comparisons of HRQoL between different types of lymphomas. Table S2: Relationship between HRQoL and respondents’ type adjusted by socioeconomic characteristics and health status. Table S3: HRQoL stratified by subtype of NHLs (A-NHL).
