# Supplementary material for: Patient–Proxy Agreement Regarding Health-Related Quality of Life in Survivors with Lymphoma: A Propensity-Score Matching Analysis
Source: Cancers (Basel). 2022 Jan 25;14(3):607. doi: 10.3390/cancers14030607 (PMC8833321; doi:10.3390/cancers14030607)
Supplement: Supplementary file 1 [file cancers-14-00607-s001.zip › cancers-1537968-supplementary.pdf]

Table S1. Comparisons of HRQoL between different types of lymphomas.

	Global			Physical			Role		
		95% C.I.			95% C.I.			95% C.I.	
	Mean	Lower limit	Upper limit	Mean	Lower limit	Upper limit	Mean	Lower limit	Upper limit
HL	62.99	60.64605	65.3241	79.58	77.54043	81.62375	76.12	73.3021	78.9367
A-NHL	60.34	59.05771	61.62057	74.67	73.45514	75.88242	67.3	65.59703	69.00313
I-NHL	61.74	60.1221	63.34865	80.67	79.43518	81.91234	77.37	75.5809	79.16201
	Emotional			Cognitive			Social		
		95% C.I.			95% C.I.			95% C.I.	
	Mean	Lower limit	Upper limit	Mean	Lower limit	Upper limit	Mean	Lower limit	Upper limit
HL	66.27	63.7299	68.80741	76.62	74.32538	78.90845	51.24	48.08361	54.40395
A-NHL	65.31	63.97935	66.64879	75.51	74.33808	76.67802	47.31	45.58835	49.02765
I-NHL	65.02	63.27196	66.75907	77.64	76.18581	79.08901	55.19	52.97944	57.3929
	Fatigue			Nausea			Pain		
		95% C.I.			95% C.I.			95% C.I.	
	Mean	Lower limit	Upper limit	Mean	Lower limit	Upper limit	Mean	Lower limit	Upper limit
HL	42.65	40.03514	45.27166	13.48	11.13112	15.83405	19.3	16.94626	21.6607
A-NHL	46.11	44.74905	47.46526	13.62	12.4472	14.78953	22.93	21.65713	24.21249
I-NHL	40.57	38.97478	42.1718	8.84	7.62962	10.05655	19.95	18.38262	21.511
	Dyspnea			Insomnia			Appetite		
		95% C.I.			95% C.I.			95% C.I.	
	Mean	Lower limit	Upper limit	Mean	Lower limit	Upper limit	Mean	Lower limit	Upper limit
HL	39.7	36.98264	42.42035	26.77	23.7215	29.81084	23.58	20.6372	26.52698
A-NHL	42.91	41.49208	44.33531	30.75	29.13818	32.35562	26.47	24.96043	27.9823
I-NHL	39.05	37.36369	40.73915	31.16	29.10439	33.2183	20.79	19.02759	22.55042
	Constipation			Diarrhea					
		95% C.I.			95% C.I.				
	Mean	Lower limit	Upper limit	Mean	Lower limit	Upper limit			
HL	17.21	14.4403	19.98756	10.55	8.385861	12.70867			
A-NHL	18.03	16.62801	19.42371	13.64	12.49066	14.79886			
I-NHL	16.45	14.74594	18.14413	12.54	11.14116	13.94749			

Table S2. Relationship between HRQoL and respondents' type adjusted by socioeconomic characteristics and health status.

	**Physical**			**Role**		
	**Model 1**	**Model 2**	**Model 3**	**Model 4**	**Model 5**	**Model 6**
	**Full model**	**Being treated model**	**Treatment completed model**	**Full model**	**Being treated model**	**Treatment completed model**
Proxy	4.73(2.36,7.1)	5.54(2.02,9.07)	3.41(0.35,6.46)	2.25(-1.12,5.61)	3.22(-1.85,8.29)	0.63(-3.61,4.88)
Female	-3.72(-6.06,-1.38)	-3.57(-7.09,-0.05)	-3.72(-6.71,-0.74)	-0.07(-3.4,3.25)	-1.73(-6.79,3.33)	2.02(-2.13,6.16)
41-60	-3.58(-6.36,-0.79)	-3.33(-7.47,0.8)	-3.73(-7.37,-0.09)	-4.47(-8.42,-0.52)	-4.72(-10.66,1.22)	-3.94(-9,1.11)
≥61	-8.59(-14.11,-3.07)	-5.61(-13.55,2.34)	-15.11(-22.61,-7.61)	-4.13(-11.95,3.7)	-2.09(-13.51,9.33)	-8.95(-19.37,1.47)
Tertiary and above	-2.44(-5.17,0.29)	-2.77(-6.82,1.28)	-1.66(-5.19,1.88)	-3.29(-7.16,0.58)	-3.77(-9.6,2.06)	-1.9(-6.82,3.01)
Rural resident	-0.41(-3.71,2.89)	-1.92(-6.69,2.85)	1.91(-2.53,6.34)	-4.18(-8.86,0.5)	-6.46(-13.33,0.4)	0.14(-6.02,6.31)
Married	0.97(-4.11,6.04)	-1.9(-9.56,5.75)	4.78(-1.65,11.21)	3.34(-3.86,10.54)	2.74(-8.26,13.75)	4.09(-4.84,13.03)
Divorce/widow(er)	3.54(-2.8,9.89)	1.82(-7.87,11.51)	5.92(-2.03,13.87)	7.98(-1.02,16.98)	7.37(-6.56,21.29)	8.71(-2.34,19.76)
50,001~100,000	5.72(2.46,8.97)	5.11(0.21,10.02)	6.07(1.95,10.18)	2.27(-2.34,6.89)	0.72(-6.33,7.77)	3.8(-1.91,9.52)
≥100,001	3.45(0.8,6.1)	4.28(0.38,8.17)	2.09(-1.37,5.55)	1.75(-2,5.5)	2.33(-3.27,7.92)	0.77(-4.04,5.57)
Non-employed	-4.19(-6.93,-1.44)	-3.77(-7.91,0.37)	-4.97(-8.45,-1.49)	-6.93(-10.82,-3.03)	-7.35(-13.3,-1.39)	-6.73(-11.56,-1.89)
Retired	0.57(-3.49,4.63)	0.79(-5.49,7.06)	0.94(-4.08,5.96)	3.58(-2.18,9.34)	4.03(-5,13.05)	3.37(-3.61,10.34)
URBS	1.46(-3.69,6.61)	0.66(-7.44,8.75)	3.25(-2.97,9.46)	0.98(-6.32,8.29)	-2.22(-13.85,9.42)	5.4(-3.24,14.03)
NRCS	0.85(-2.61,4.3)	-1.35(-6.38,3.67)	3.96(-0.6,8.52)	3.38(-1.52,8.28)	1.85(-5.38,9.07)	5.9(-0.44,12.23)
FMS	2.74(-1.04,6.52)	4.41(-1.16,9.97)	-0.04(-4.98,4.9)	5.15(-0.21,10.52)	8.46(0.46,16.46)	-0.19(-7.05,6.67)
Duration	0.09(-0.64,0.82)	-0.1(-1.79,1.6)	0.18(-0.53,0.89)	0.51(-0.53,1.55)	0.44(-2,2.88)	0.57(-0.42,1.55)
Chemotherapy, Yes	-2.66(-7.19,1.87)	-1.76(-8.05,4.53)	-2.52(-9.25,4.21)	-6.35(-12.78,0.08)	-7.07(-16.11,1.97)	-3.78(-13.13,5.58)

Immunotherapy, Yes	1.32(-1.04,3.67)	2.12(-1.35,5.6)	-0.63(-3.71,2.46)	2.12(-1.21,5.46)	2.09(-2.9,7.09)	1.37(-2.92,5.65)
Radiotherapy, Yes	1.17(-1.54,3.88)	1.68(-2.78,6.14)	0.32(-2.84,3.48)	3.75(-0.09,7.6)	5.95(-0.47,12.36)	1.56(-2.83,5.95)
Surgery, Yes	-3.68(-6.78,-0.59)	-5.59(-10.7,-0.48)	-2.2(-5.78,1.39)	-5.34(-9.73,-0.95)	-6.52(-13.86,0.82)	-4.61(-9.6,0.37)
Being treated	-11.42(-13.72,-9.12)			-21.81(-25.07,-18.54)		

Table S2. *Cont.*

	Emotional			Cognitive		
	**Model 7**	**Model 8**	**Model 9**	**Model 10**	**Model 11**	**Model 12**
	**Full model**	**Being treated model**	**Treatment completed model**	**Full model**	**Being treated model**	**Treatment completed model**
Proxy	1.46(-1.39,4.32)	3.06(-1,7.13)	3.06(-1,7.13)	-3.98(-6.53,-1.42)	-3.22(-6.79,0.35)	-5.41(-9.1,-1.73)
Female	-2.28(-5.1,0.54)	0.75(-3.31,4.81)	0.75(-3.31,4.81)	-3.86(-6.38,-1.33)	-3.11(-6.68,0.45)	-5.12(-8.71,-1.52)
41-60	-2.13(-5.49,1.22)	-4.84(-9.62,-0.07)	-4.84(-9.62,-0.07)	-2.65(-5.65,0.36)	-3(-7.19,1.19)	-2.98(-7.36,1.41)
≥61	3.15(-3.49,9.8)	3.52(-5.65,12.69)	3.52(-5.65,12.69)	0.48(-5.46,6.43)	3.11(-4.94,11.16)	-3.99(-13.03,5.06)
Tertiary and above	-1.11(-4.39,2.17)	0.84(-3.84,5.52)	0.84(-3.84,5.52)	-2.42(-5.36,0.52)	-2.08(-6.19,2.02)	-2.77(-7.04,1.5)
Rural resident	0.54(-3.44,4.51)	-1.4(-6.91,4.11)	-1.4(-6.91,4.11)	2.25(-1.3,5.81)	0.86(-3.98,5.69)	4.25(-1.09,9.6)
Married	-3.22(-9.33,2.89)	-5.44(-14.27,3.4)	-5.44(-14.27,3.4)	1.5(-3.97,6.97)	3.72(-4.04,11.47)	-1.32(-9.07,6.43)
Divorce/widow(er)	0(-7.64,7.63)	-3.05(-14.22,8.13)	-3.05(-14.22,8.13)	3.33(-3.51,10.16)	8.38(-1.43,18.19)	-2.9(-12.49,6.69)
50,001~100,000	6.81(2.9,10.73)	4.37(-1.29,10.03)	4.37(-1.29,10.03)	5.68(2.17,9.19)	5.11(0.14,10.08)	5.95(0.99,10.91)
≥100,001	2.82(-0.36,6.01)	3.7(-0.79,8.19)	3.7(-0.79,8.19)	1.44(-1.41,4.29)	2.34(-1.6,6.29)	0.02(-4.16,4.19)
Non-employed	-4.32(-7.62,-1.01)	-4.89(-9.67,-0.11)	-4.89(-9.67,-0.11)	-4.88(-7.84,-1.93)	-4.98(-9.18,-0.79)	-4.94(-9.14,-0.75)
Retired	0.68(-4.21,5.57)	-1.65(-8.89,5.6)	-1.65(-8.89,5.6)	-2.97(-7.34,1.41)	-4.35(-10.71,2.01)	-1.59(-7.64,4.46)
URBS	3.27(-2.93,9.47)	4.69(-4.65,14.03)	4.69(-4.65,14.03)	1.84(-3.7,7.39)	3.05(-5.15,11.25)	1.64(-5.86,9.13)
NRCS	1.06(-3.09,5.22)	0.95(-4.85,6.76)	0.95(-4.85,6.76)	-0.73(-4.45,2.99)	-2.69(-7.78,2.4)	2.43(-3.07,7.92)
FMS	2(-2.55,6.55)	3.91(-2.51,10.33)	3.91(-2.51,10.33)	-0.54(-4.62,3.53)	3.17(-2.47,8.81)	-6.33(-12.29,-0.38)
Duration	0.02(-0.86,0.9)	0.49(-1.47,2.45)	0.49(-1.47,2.45)	-0.06(-0.85,0.72)	-0.29(-2,1.43)	-0.02(-0.87,0.83)
Chemotherapy, Yes	-4.18(-9.63,1.28)	-6.56(-13.82,0.7)	-6.56(-13.82,0.7)	-4.17(-9.05,0.72)	-6.29(-12.66,0.09)	0.31(-7.81,8.43)

Immunotherapy, Yes	0.13(-2.7,2.96)	-2.21(-6.22,1.8)	-2.21(-6.22,1.8)	0.45(-2.08,2.99)	-0.3(-3.82,3.22)	0.9(-2.81,4.62)
Radiotherapy, Yes	0.12(-3.14,3.38)	-0.27(-5.42,4.88)	-0.27(-5.42,4.88)	-1.37(-4.28,1.55)	-1.66(-6.18,2.86)	-1.04(-4.85,2.76)
Surgery, Yes	-3.39(-7.12,0.33)	-3.87(-9.77,2.02)	-3.87(-9.77,2.02)	-1.81(-5.15,1.52)	-2.6(-7.78,2.57)	-1.51(-5.83,2.82)
Being treated	-12.62(-15.39,-9.85)			-6.98(-9.46,-4.5)		

Table S2. *Cont.*

	Social		
	Model 13	Model 14	Model 15
	Full model	Being treated model	Treatment completed model
Proxy	-5.09(-8.62,-1.55)	-1.58(-6.37,3.22)	-9.43(-14.74,-4.13)
Female	0.06(-3.43,3.55)	0.96(-3.83,5.75)	-0.7(-5.88,4.48)
41-60	-6.17(-10.32,-2.02)	-5.21(-10.84,0.42)	-7.71(-14.02,-1.39)
≥61	1.77(-6.45,9.99)	3.57(-7.25,14.39)	-1.76(-14.78,11.25)
Tertiary and above	-3.61(-7.67,0.45)	-3.69(-9.2,1.83)	-3.29(-9.43,2.85)
Rural resident	-1.65(-6.57,3.26)	-3.57(-10.07,2.93)	1.36(-6.34,9.06)
Married	2.21(-5.36,9.77)	0.12(-10.31,10.54)	5.47(-5.69,16.63)
Divorce/widow(er)	1.08(-8.37,10.54)	-0.38(-13.57,12.81)	2.36(-11.44,16.16)
50,001~100,000	11.52(6.66,16.37)	9.28(2.6,15.96)	13.5(6.36,20.64)
≥100,001	8.22(4.27,12.16)	7.32(2.02,12.62)	8.6(2.6,14.61)
Non-employed	-5.98(-10.07,-1.89)	-4.1(-9.74,1.54)	-8.49(-14.53,-2.45)
Retired	1.18(-4.87,7.23)	0.13(-8.42,8.67)	2.11(-6.6,10.82)
URBS	4.36(-3.31,12.03)	2.86(-8.16,13.89)	7(-3.79,17.78)
NRCS	0.78(-4.36,5.93)	-1.86(-8.7,4.98)	4.58(-3.33,12.5)
FMS	-0.81(-6.45,4.82)	-0.62(-8.19,6.96)	-2.84(-11.41,5.73)
Duration	0.39(-0.7,1.48)	1.52(-0.79,3.83)	0.04(-1.19,1.27)
Chemotherapy, Yes	-6.52(-13.27,0.24)	-11.85(-20.41,-3.28)	7.16(-4.52,18.85)
Immunotherapy, Yes	0.37(-3.13,3.88)	-0.19(-4.92,4.54)	0.23(-5.12,5.58)
Radiotherapy, Yes	3.11(-0.92,7.15)	2.66(-3.42,8.73)	3.67(-1.81,9.15)
Surgery, Yes	-5.4(-10.02,-0.79)	-6.71(-13.66,0.25)	-3.26(-9.48,2.97)
Being treated	-16.82(-20.25,-13.39)		

Table S3. HRQoL stratified by subtype of NHLs (A-NHL).

	Global		physical		role		emotional		cognitive		social	
	mean	sd	mean	sd	mean	sd	mean	sd	mean	sd	mean	sd
BL	55.09	22.47	67.59	31.07	56.94	38.7	56.94	27.42	71.76	19.85	40.74	34.38
DLBLC	62.03	23.39	75.8	22.06	69.42	30.29	66.42	24.48	75.6	21.86	50.16	31.5
ENKL	62.56	24.1	78.51	19.15	68.59	31.58	68.85	24.35	78.97	17.68	45.9	30.64
MCL	56.14	20.56	74.59	19.34	67	27.8	60.47	22.51	72.86	21.93	42.79	29.04
PTCL	53.03	22.15	66.26	21.59	56.06	31.44	64.02	22.93	77.02	18.89	39.65	28.46
T-LBL	54.67	23.58	65.56	22.48	56.06	33.16	61.36	21.11	73.99	20.28	36.11	29.03
p-value	<0.001		<0.001		<0.001		0.005		0.18		<0.001	
	Fatigue		Nausea		Pain		Dyspnea		Insomnia		Appetite	
	mean	sd	mean	sd	mean	sd	mean	sd	mean	sd	mean	sd
BL	50	26.63	16.67	23.9	26.39	30.44	47.22	30.21	31.48	29.76	33.33	29.81
DLBLC	44.27	24.74	11.97	19.76	22.22	23.06	41.21	25.66	30.03	29.22	23.79	26.63
ENKL	44.19	24.06	15.77	23.14	21.28	21.96	42.31	24.83	25.9	29.13	29.23	29.64
MCL	51.88	23.63	16.22	25.64	23.09	22.46	48.65	25.31	38.29	30.21	29.73	26.69
PTCL	51.68	22.94	15.91	19.49	27.27	24.04	46.97	24.79	33.33	26.79	33.33	26.15
T-LBL	52.02	23.88	19.95	22.5	28.54	22.41	45.96	26.64	29.29	25.83	36.36	29.08
p-value	<0.001		0.007		0.13		0.01		0.01		<0.001	
	Constipation		Diarrhea									
	mean	sd	mean	sd								
BL	20.37	24.27	20.37	22.93								
DLBLC	18.4	25.99	12.08	19.55								
ENKL	14.36	23.46	12.82	19.19								
MCL	18.24	25.3	17.57	25.63								
PTCL	17.17	24.28	20.71	22.47								
T-LBL	19.7	22.63	15.15	23.52								
p-value	0.6		<0.001									

Table S3. *Cont.* (I-NHL)

	Global		physical		role		emotional		cognitive		social	
	mean	sd	mean	sd	mean	sd	mean	sd	mean	sd	mean	sd
CLL	60.64	22.27	80.21	17.28	76.77	24.88	62.23	23.72	76.6	19.4	55.23	30.01
FL	62.77	21.84	81.97	15.96	78.41	23.76	65.79	24.18	79.09	18.73	56.29	30.72
LPL	57.1	20.57	71.23	22.31	69.75	31.9	65.9	24.51	73.46	24.98	51.85	30.49
MALT	61.29	24.15	82	17.69	79.09	23.73	63.56	26.15	76.97	22	53.48	32.57
PC-ALCL	64.37	26.2	80.8	17.17	77.01	27.72	71.41	23.58	77.59	23.68	54.89	32.3
p-value	0.37		<0.001		0.18		0.12		0.34		0.84	
	Fatigue		Nausea		Pain		Dyspnea		Insomnia		Appetite	
	mean	sd	mean	sd	mean	sd	mean	sd	mean	sd	mean	sd
CLL	41.19	22.82	7.45	15.44	21.28	22.26	40.25	24	31.74	29.51	21.1	24.58
FL	39.44	22	8.77	16.72	17.59	20.58	37.82	23.38	32.26	28.69	20.27	23.36
LPL	44.44	22.22	9.57	18.77	26.23	24.36	41.98	21.66	23.46	26.41	24.07	29.97
MALT	39.6	20.99	8.03	15.92	21.52	21.1	38.79	24.12	32.12	28.17	19.7	24.02
PC-ALCL	43.49	25.51	14.66	22.09	20.69	25.8	40.23	24.79	28.16	29.82	21.84	28.31
p-value	0.43		0.08		0.05		0.66		0.27		0.83	
	Constipation		Diarrhea									
	mean	sd	mean	sd								
CLL	14.54	22.37	10.99	18.17								
FL	17.25	24.31	12.96	19.71								
LPL	17.9	24.84	13.58	19.98								
MALT	14.24	20.92	12.42	20.62								
PC-ALCL	20.69	28.5	14.37	21.73								
p-value	0.33		0.74									
